# Supplementary material for: Study on flow field characteristics of gas-liquid hydrocyclone separation under vibration conditions
Source: PLoS One. 2024 Jul 12;19(7):e0307110. doi: 10.1371/journal.pone.0307110 (PMC11244842; doi:10.1371/journal.pone.0307110)
Supplement: Attachment — Submitted filename: Response to Reviewers.docx [file pone.0307110.s002.docx]

-------------------------------------------------------------------------------------------------------

**Response to the Reviewer #1’s Comments: PONE-D-24-11282**

-------------------------------------------------------------------------------------------------------

Manuscript PONE-D-24-11282

“Study on flow field characteristics of gas-liquid hydrocyclone separation under vibration conditions”

We thank the reviewers for the helpful comments, which help improve the quality of the paper. Below, reviewers’ comments are presented with *italics* to distinguish from our response. We also submit a Word copy of the revised manuscript in which important revisions are highlighted for easy identification.

## Reviewer #1:

This article must be strictly revised by considering the following comments.

**Comment** 1) *For abstract, the authors did not inform the reader that computational fluid dynamics was used as a tool for the authors’ work. So, the reviewer suggests the authors to add the comprehensive and informative abstract in the revised manuscript.*

**Response to comment 1:** We thank the reviewer for the helpful comment. We have added the research tools used in the article to the abstract.

**Revision:** On Page 1, Paragraph 1 of the “Abstract” section, Line 4-7, the revised text reads, “Based on Computational Fluid Dynamics (CFD), Computational Solid Mechanics Method (CSM) and fluid-solid coupling method, a fluid-solid coupling mechanical model of a gas-liquid cyclone is established.”

**Comment** 2) *From Figure 1, the reviewer understands that the hydrocyclone is horizontally located. Does the reviewer understand your considered hydrocyclone correctly? Please clearly describe your considered hydrocyclone system in the revised manuscript.*

**Response to comment 2:** We thank the reviewer for the helpful comment. For the actual use of hydrocyclone systems, both horizontal and vertical positioning are feasible in terms of structure. In actual working conditions, the impact of centrifugal force is much greater than gravity. The description of the hydrocyclone systems is detailed in the first paragraph of section 2.1.

**Comment** 3) *The authors only showed the schematic of the considered hydrocyclone. However, there is no information of the dimensions of the considered hydrocyclone. Please report all details of the considered hydrocyclone in the revised manuscript.*

**Response to comment 3:** We thank the reviewer for the helpful comment. We have added information on the structural dimensions of the hydrocyclone, as shown in Figure 1b) and Table 1.

**Revision:** On Page 4, Paragraph 2 of section 2.1, the revised text reads, “The main size parameters of the gas-liquid separator structure are shown in Figure 1b). The main diameter *D* is 96mm, the number of spiral guide blades is set to 5, the length of the gas phase flow channel is *L*_1_, the inner diameter of the gas phase outlet is *D*_1_, the height of the bottom flow inverted cone is *L*_2_, the inner diameter of the liquid phase outlet is *D*_2_, the pitch is *L*_3_, the inner diameter of the bottom flow inverted cone is *D*_3_, and the length of the main body of the separator is *L*_4_. The main structural parameters of each part of the initial structure are shown in the figure 1b), and the specific dimensions of the structural parameters of each part are shown in Table 1. Each parameter is expressed as a multiple of the main diameter *D*.” Added new Figure 1b) and Table 1 in Section 2.1 of the article.

**Comment** 4) *The authors used Reynolds stress turbulence model for this work. However, the pressure-strain sub-model was not reported. Please report the pressure-strain sub-model in the revised manuscript.*

**Response to comment 4:** We thank the reviewer for the helpful comment. The pressure-strain sub-model adopts a linear strain model.

**Revision:** On Page 5, Paragraph 2 of the “2.2.1 Fluid governing equation” section, Line 3-4, the revised text reads, “Pressure-strain sub-model adopts linear strain.”

**Comment** 5) *The authors must also report the selected wall functions of the present work.*

**Response to comment 5:** We thank the reviewer for the helpful comment. The wall function uses the standard wall functionand.

**Revision:** On Page 7, Paragraph 1 of the “2.4 boundary condition setting” section, Line 4, the revised text reads, “the wall function uses the standard wall functionand.”

**Comment** 6) *The authors only showed the information on an algorithm (Coupled) used in the present work. However, the information of spatial and temporal discretization schemes was absent. Therefore, in the revised manuscript, the spatial and temporal discretization schemes used in the present work must be reported. Moreover, please make the discussion between the selected set of numerical schemes of this work and the suggested numerical scheme set of the previous work (10.1016/j.powtec.2023.118713)*.

**Response to comment 6:** We thank the reviewer for the helpful comment. We have added the spatial and temporal discretization schemes used in the present work. We also referred to the literature content (10.1016/j.powtec.2023.118713).

**Revision:** On Page 7, Paragraph 1 of the “2.4 boundary condition setting” section, Line 11-13, the revised text reads, “QUICK scheme with second-order precision is used for the dispersion of momentum equation, second-order upwind scheme is used for turbulent kinetic energy and dissipation rate.” Added reference [26] in the references section.

**Comment** 7) *Please compare and show the residence time and the selected time step size of 0.005 s in the revised manuscript to confirm that the selected time step size is appropriate for the present simulations (Generally, the time step size should be shorter than 1/100 of residence time)*.

**Response to comment 7:** We thank the reviewer for the helpful comment. The characteristic size of the calculation model in this article is 30mm, with an average speed of 0.6m/s, resulting in a residence time of 0.05s. The general time step is one to two orders of magnitude smaller than the dwell time, so 0.005s is chosen as the time step in this article. In addition, under the condition of using this time step, the physical parameters of interest have reached a convergence state, as shown in the monitoring curve of the air mass flow rate at the overflow port in Fig. R1 below. Therefore, the selected time step is suitable for the current simulation.

Figure R1 Monitoring curve of air quality flow rate at overflow port

**Comment** 8) *The authors did not show the convergence criterion for the present simulations. Please show the information on convergence criterion in the revised manuscript.*

**Response to comment 8:** We thank the reviewer for the helpful comment. In this article, the accuracy of the residual is controlled to 1×10^-6^ during simulation. During the iterative solution process, the residual of each iteration is calculated, which is the difference between the current iteration solution and the previous iteration solution. When the residual drops to a preset threshold less than a certain threshold, it is considered that the calculation has converged.

**Revision:** On Page 7, Paragraph 1 of the “2.4 boundary condition setting” section, Line 13-14, the revised text reads, “and the accuracy of the residual is controlled to 1x10^-6^.”

**Comment** 9) *Please represent the governing equations used in the present work in the revised manuscript.*

**Response to comment 9:** We thank the reviewer for the helpful comment. The corresponding formulas (1)-(5) have been added, and the fluid microelements in the hydrocyclone should satisfy the conservation of mass and momentum. The mass and momentum control equations in the fluid domain can be found in formulas (1) and (2), respectively. In order to simulate the characteristics of gas-liquid separation flow field more accurately, a more accurate Reynolds stress model (RSM) is used to complete the turbulence calculation in the flow field. The pressure-strain sub-model adopts linear strain, and the expression of the Reynolds stress transport equation is shown in formula (3). The motion equation of the structure is discretized using the finite element method during fluid structure coupling analysis, taking into account the combined effect of internal and external forces in the pipe column structure. The dynamic equation of the structure is shown in formula (4).

**Revision:** On Page 4, the “2.2.1 Fluid governing equation “and “2.2.2 Dynamic equation of tubular string structure” section, the revised text reads, “2.2.1 Fluid governing equation

The fluid microelements in the hydrocyclone should satisfy the conservation of mass and momentum. The governing equations of mass and momentum in the fluid domain are:

$\frac{\partial\rho}{\partial t}+div(\rho u_{i})=0$ (1)

$\frac{\partial\rho u_{i}}{\partial t}+\frac{\partial}{\partial x_{j}}(\rho u_{i}u_{j})=-\frac{\partial p}{\partial x_{i}}+\frac{\partial}{\partial x_{j}}(\mu\frac{\partial u_{i}}{\partial x_{j}}-\rho\overline{u_{i}^{'}u_{j}^{'}})$ (2)

In order to more accurately simulate the characteristics of the gas-liquid separation flow field, a more accurate Reynolds stress model (RSM) is used to complete the turbulence calculation in the flow field. Pressure-strain sub-model adopts linear strain. The expression of the Reynolds stress transport equation is:

 (3)

2.2.2 Dynamic equation of tubular string structure

The motion equation of the structure is that in the fluid-structure coupling analysis, the finite element method is used to discrete the structure, considering the joint action of the internal and external forces of the pipe string structure, and the dynamic equation of the structure is:

 (4)

”

On Page 5, Paragraph 2 of the “2.3 Basic parameters and settings” section, Line 3-4, the revised text reads, “[24]. See Equations (5) and (6) for the coalescence kernel and fragmentation kernel functions.”

On Page 5, Paragraph 3-4 of the “2.3 Basic parameters and settings” section, the revised text reads, “

“$a\left(V_{i}\text{、}V_{j}\right)=\omega ag\left(V_{i}\text{、}V_{j}\right)Pag\left(V_{i}\text{、}V_{j}\right)\left[m^{3}/\sec\right]$ (5)

$\Omega br\left(V\text{、}V'\right)=K\int_{\xi_{\min}}^{1} \frac{\left(1+\xi\right)^{2}}{\xi^{n}}\exp\left(-b\xi^{m}\right)d\xi$ (6)

”

**Comment** 10) *The authors used mesh-based CFD model for the present work. However, the details of grid generation and figure of grid generation used in the present work were absent. Please add information of grid generation of the considered hydrocyclone in the revised manuscript.*

**Response to comment 10:** We thank the reviewer for the helpful comment. The wall surface of the hydrocyclone is a rigid non slip wall, and its structure is linear elastic within a small deformation range. There is only static friction between the fluid and the wall of the hydrocyclone, and the remaining damping is ignored. The mesh of the structural domain is also divided into tetrahedral meshes, and the schematic diagram of the division results is shown in figure 2 a).

**Revision:** On Page 3, Paragraph 3 of the section 2.1, Line 1-5, the revised text reads, “The wall of the swirl separation string is a rigid non-slip wall, and the wall of the swirl separation string is linearly elastic in a small deformation range. There is only mutual static friction between the fluid and the swirl separation string, and the rest of the damping is ignored. The mesh of the fluid domain is also divided by a tetrahedral mesh. The schematic diagram of the division results is shown in the figure 2 a).”

**Comment** 11) *In the present work, the authors did not show grid independence study, which is an important section of the CFD article, in the present manuscript. The authors must add grid independence study in the revised manuscript.*

**Response to comment 10:** We thank the reviewer for the helpful comment. We have conducted an independence check on the grid before officially starting the simulation calculation of the article, and this section has been added to the revised draft.

**Revision:** On Page 4, Paragraph 3 of the section 2.1, Line 6-14, the revised text reads, “and the independence test of the mesh is carried out. The number of mesh elements corresponding to different division levels 1 to 5 is 650736, 846224, 1035930, 1255916, 1496220, respectively. The volume fraction of gas in the bottom stream can directly reflect the water removal performance of the gas-liquid separator, so the volume fraction of gas in the bottom stream is used as the test standard. The grid independence verification is shown in figure 2b). The graphs corresponding to levels 3 to 5 show that the gas volume fraction of the underflow is relatively stable, and the final decision is based on the accuracy of the equilibrium numerical simulation and the calculation time, and the meshing level of 1035930 is adopted.”

**Comment** 12) *For validation, the authors validated the model by considering the collection efficiency. However, the unknown parameters for the RANS equations are mean pressure and velocities. Hence, it is better to validate the present CFD model by comparing the simulated velocity profiles with the measured profiles. Please describe why the author considered collection efficiency for model validation or show the model validation by using the velocity profile comparison.*

**Response to comment 12:** We thank the reviewer for the helpful comment. From the currently available research, it can be seen that only the effectiveness data validates the model, and there are currently no other validation data available.

**Comment** 13) *For velocity profile comparisons, the horizontal axis labels were dimensionless radial distance (r/R). At first sight, these horizontal axis labels are seemingly proper for velocity profile comparisons. However, the velocity profiles for swirl flow inside cyclone separators are not symmetrical. Therefore, the authors must use dimensionless distance (e.g., x/R for section S2 of Figure 2) for velocity profile comparisons in the revised manuscript.*

**Response to comment 13:** We thank the reviewer for the helpful comment. The *r*/*R* in this article is equivalent to *x*/*R*, both representing dimensionless radial distance.

**Comment** 14) *Color map for contour of Figure 15 was not presented. Please add it into the Figure 15 of the revised manuscript.*

**Response to comment 14:** We thank the reviewer for the helpful comment. We have added a color map for contour of figure 15((updated to figure 16 in the revised manuscript).)

**Revision:** Figure 16.

**Comment** 15) *Typing errors and English grammar must be checked.*

**Response to comment 15:** We thank the referee for the helpful comment. We have made an overall check of the manuscript to correct the Typing errors and English grammar.

## Reviewer #2:

In this study, the authors used external volume force as vibrational energy to improve gas-liquid separation efficiency. However, some comments are regarding the numerical modeling of multiphase flow in gas-liquid hydrocyclone device.

Comments are given below:

**Comment** *1. In the Introduction section, please cite the reference related to the Computational Fluid Dynamics method (CFD). Is it related to the VOF method?*

**Response to comment 1:** We thank the reviewer for the helpful comment. Relevant literature [18-20] has been cited in the introduction section, confirming that the method used in this article is independent of VOF

**Revision:** On Page 2, Paragraph 2 of the “1 Introduction” section, Line 17-26, the revised text reads, “Li S et al. [18] compared and analyzed the velocity field, pressure field and turbulent kinetic energy distribution in the variable diameter circular tube of a hydrocyclone under coupled and uncoupled conditions, and found that the influence of coupling on the flow field in the structure cannot be ignored. Xu Y et al. [19] studied the fluid-solid coupling of the spiral flow in the cyclone separator under vibration conditions, established a two-way fluid-solid coupling model of the cyclone under vibration conditions, and found that the movement of the structure caused the migration of the flow field structure. Li S et al. [20] found that the separation efficiency of the hydrocyclone under vibration coupling conditions is lower than that under uncoupled conditions.”

“

18.	Li S，ZHANG J ，WANG Z C. Numerical simulation of hydrocyclone’s inner flow field under fluid structure interaction[J]. Chemical Engineering and Machinery, 2015, 42(05):706-710.

19.	XU Y，ZHANG Y Y，XU D K，et al．Study on fluid-structure interaction in hydrocyclone under vibration[J]. Journal of China University of Petroleum(Edition of Natural Science)，2017，41(4):140-147．

20.	Li S, Li R, Nicolleau FCGA Study on oil–water two-phase flow characteristics of the hydrocyclone under periodic excitation[J]. Chemical Engineering Research and Design, 2020, 159: 215-224.

”

**Comment** *2. In the introduction section, please cite the references related to the computational solid mechanics method, fluid-structure coupling theory, and fluid-structure coupling mechanical model.*

**Response to comment 2:** We thank the reviewer for the helpful comment. We have added and cited three references related to computational solid mechanics methods, fluid structure coupling theory, and fluid structure coupling mechanics models [18-20].

**Revision:** On Page 2, Paragraph 2 of the “1 Introduction” section, Line 17-26, the revised text reads, “Li S et al. [18] compared and analyzed the velocity field, pressure field and turbulent kinetic energy distribution in the variable diameter circular tube of a hydrocyclone under coupled and uncoupled conditions, and found that the influence of coupling on the flow field in the structure cannot be ignored. Xu Y et al. [19] studied the fluid-solid coupling of the spiral flow in the cyclone separator under vibration conditions, established a two-way fluid-solid coupling model of the cyclone under vibration conditions, and found that the movement of the structure caused the migration of the flow field structure. Li S et al. [20] found that the separation efficiency of the hydrocyclone under vibration coupling conditions is lower than that under uncoupled conditions.”

“

18. Li S，ZHANG J ，WANG Z C. Numerical simulation of hydrocyclone’s inner flow field under fluid structure interaction[J]. Chemical Engineering and Machinery, 2015, 42(05):706-710.

19. XU Y，ZHANG Y Y，XU D K，et al．Study on fluid-structure interaction in hydrocyclone under vibration[J]. Journal of China University of Petroleum(Edition of Natural Science)，2017，41(4):140-147．

20. Li S, Li R, Nicolleau FCGA Study on oil–water two-phase flow characteristics of the hydrocyclone under periodic excitation[J]. Chemical Engineering Research and Design, 2020, 159: 215-224.

”

**Comment** *3. In the models and method section, please mention the dimensions of the schematic figure in the Figure 1 caption.*

**Response to comment 3:** We thank the reviewer for the helpful comment. We have added information on the structural dimensions of the hydrocyclone, as shown in Figure 1b) and Table 1.

**Revision:** On Page 4, Paragraph 2 of section 2.1, the revised text reads, “The main size parameters of the gas-liquid separator structure are shown in Figure 1b). The main diameter *D* is 96mm, the number of spiral guide blades is set to 5, the length of the gas phase flow channel is *L*_1_, the inner diameter of the gas phase outlet is *D*_1_, the height of the bottom flow inverted cone is *L*_2_, the inner diameter of the liquid phase outlet is *D*_2_, the pitch is *L*_3_, the inner diameter of the bottom flow inverted cone is *D*_3_, and the length of the main body of the separator is *L*_4_. The main structural parameters of each part of the initial structure are shown in the figure 1b), and the specific dimensions of the structural parameters of each part are shown in Table 1. Each parameter is expressed as a multiple of the main diameter *D*.” Added new Figure 1b) and Table 1 in Section 2.1 of the article.

**Comment** *4. In the fluid-structure coupling model, please mention the version of the ANSYS workbench that was used to perform the numerical simulation.*

**Response to comment 4:** We thank the reviewer for the helpful comment. The version of ANSYS Workbench used for numerical simulation in this article is 2022R1.

**Revision:** On Page 4, Paragraph 1 of the “2.2 Fluid-structure coupling mechanical model” section, Line 1-2, the revised text reads, “The ANSYS Workbench 2022R1 is used to establish the mechanical model of the downhole gas-liquid hydrocyclone ”

**Comment** *5. In the one-way coupling (where fluid dynamics are accounted for), please explain the major assumptions of the numerical simulations.*

**Response to comment 5:** We thank the reviewer for the helpful comment. This article uses a twin-screw pump system to suction the outlet of the hydrocyclone separation column. To facilitate subsequent research, 5 additional hypotheses have been added.

**Revision:** On Page 4, Paragraph 1-6 of the “2.2 Fluid-structure coupling mechanical model” section, the revised text reads, “In this paper, a twin-screw pump system is used to pump the outlet of the swirl separation string. In order to facilitate subsequent research, the following assumptions should be made:

(1) When the single screw pump is used, other units in the well work in an ideal state, and the exciting force on the swirl separation string is only generated by the recovery pump or the injection pump;

(2) The overflow and underflow connection pipelines are homogeneous and isotropic elastic material straight rods;

(3) Ignore the influence of local stiffness and mass of flange joints on overall vibration.

(4) The boundary conditions at both ends of the model are that one end is free and the other end is fixed.

(5) The exciting force on the swirl separation string is a sinusoidal load that changes with time in one direction only in the radial direction.

”

**Comment** *6. Please cite the reference of the "Fill command" that will be useful to readers.*

**Response to comment 6:** We thank the reviewer for the helpful comment. We have supplemented the literature [21]

**Revision:** 21. Jin Y. Flow field characteristics of swirl separation string under vibration condition[J]. Chemical Engineering,2023,51(11):48-54.

**Comment** *7. Please cite the reference of "Fluid-Structure coupling interface."*

**Response to comment 7:** We thank the reviewer for the helpful comment. The interface where the structural domain directly contacts and interacts with the fluid domain is a fluid structure coupling interface, which we have supplemented with relevant literature.

**Revision:** 22. Li S, Li R, Wang Z, et al. Fluid-structure interaction vibration response analysis of the hydrocyclone under periodic excitation[J]. IEEE Access,2019, 7:146273-146281.

**Comment** *8. It would be nice if the authors could explain all essential governing equations related to the mixture CFD model, kernel mixture model with PBM, and coalescence kernel, or the authors could cite the references related to the proposed numerical methods. In this physical problem layout, the essential governing equations could be split into two parts (i.e., FSI model (where fluid mechanics and solid structure mechanics are accounted for) and No FSI model (where Turbulence model: Reynolds stress model could be discussed). Based on the essential governing, authors could discuss the boundary conditions and numerical solver settings in the material and method sections or the appendix section.*

**Response to comment 8:** We thank the reviewer for the helpful comment. The PBM coalescence and fragmentation kernel functions are shown in formulas (5) and (6)

**Revision:** On Page 5, Paragraph 2-4 of the “2.3 Basic parameters and settings” section, the revised text reads, “ “See Equations (5) and (6) for the coalescence kernel and fragmentation kernel functions.

$a\left(V_{i}\text{、}V_{j}\right)=\omega ag\left(V_{i}\text{、}V_{j}\right)Pag\left(V_{i}\text{、}V_{j}\right)\left[m^{3}/\sec\right]$ (5)

$\Omega br\left(V\text{、}V'\right)=K\int_{\xi_{\min}}^{1} \frac{\left(1+\xi\right)^{2}}{\xi^{n}}\exp\left(-b\xi^{m}\right)d\xi$ (6)

”

**Comment** *9. In the material and method sections, authors could discuss the grid generation in terms of mesh independence study, model selection and boundary conditions, and solvers (i.e., phase bound simple algorithm), respectively, or this section could be placed in the appendix section, where, all essential governing equations will be discussed.*

**Response to comment 9:** We thank the reviewer for the helpful comment. We have conducted an independence check on the grid before officially starting the simulation calculation of the article, and this section has been added to the revised draft.

**Revision:** On Page 4, Paragraph 3 of the section 2.1, Line 6-14, the revised text reads, “and the independence test of the mesh is carried out. The number of mesh elements corresponding to different division levels 1 to 5 is 650736, 846224, 1035930, 1255916, 1496220, respectively. The volume fraction of gas in the bottom stream can directly reflect the water removal performance of the gas-liquid separator, so the volume fraction of gas in the bottom stream is used as the test standard. The grid independence verification is shown in figure 2b). The graphs corresponding to levels 3 to 5 show that the gas volume fraction of the underflow is relatively stable, and the final decision is based on the accuracy of the equilibrium numerical simulation and the calculation time, and the meshing level of 1035930 is adopted.”

**Comment** *10. In the validation section, the authors validate experimental results with obtained numerical results because, in the context of the numerical methodology section, authors used water, oil, and gas as working fluid systems, but in the experimental section, authors used experimental results where two-phase (oil-water) fluid-structure interaction coupling is accounted for. We should validate similar systems.*

**Response to comment 10:** We thank the reviewer for the helpful comment. The actual working condition studied in this paper is oil-water-gas three-phase, but in view of the lack of relevant experimental data, the data of oil-water two-phase are used for verification.

**Comment** *11. In the basic parameters and settings section, authors could make a table related to all model input parameters with numerical solver settings (with convergence criteria).*

**Response to comment 11:** We thank the reviewer for the helpful comment. In this paper, the accuracy of the residual error is controlled to 1×10^-6^ during the simulation. In the iterative solution process, the residual error of each iteration is calculated, that is, the difference between the current iterative solution and the previous iterative solution. When the residual error drops to a preset less than a certain threshold, the calculation is considered to be convergent.

**Comment** *12. In the velocity field section, it would be nice if the authors could discuss axial, radial, and tangential velocity distributions with streamlined velocity vector plots at different locations of gas-liquid hydrocyclone separators. These plots will be valid for the NO FSI model and the FSI model, respectively, and discuss the significant difference between the two proposed methods (i.e., NO FSI and FSI model).*

**Response to comment 12:** We thank the reviewer for the helpful comment. The velocity line charts of different sections used in this paper can also clearly show the significant differences in axial, radial and tangential velocities under NO FSI and FSI models, so we do not add the description of vector diagrams.

**Comment** *13. In the bubble particle size section, authors could make a plot related to the separation efficiency as a function of gas bubble size with different inlet velocities of the gas phase and discuss the physical insight of the process.*

**Response to comment 13:** We thank the reviewer for the helpful comment. The NO FSI studied in this paper is consistent with the gas phase velocity used in the FSI condition, and does not involve the comparison of different gas phase velocities. Therefore, it is of little significance to draw a diagram of the relationship between bubble size and separation efficiency under different gas phase inlet velocities.

**Comment** *14. In the vortex section, authors could make one plot related to the vortex finder length as a function of the operating Reynolds number, where the operating Reynolds number is defined in terms of the velocity of the gas-liquid phase system, inlet diameter of the proposed device and physicochemical properties of the working fluid system. In addition, it would be nice if the authors could discuss the concentration field distribution of the gas-liquid mixture within the proposed device.*

**Response to comment 14:** We thank the reviewer for the helpful comment. There are not enough variables to construct the relationship of the vortex finder length and the operating Reynolds number. Yes, this is a good suggestion, perhaps in the future work, we will focus on obtaining the relationship between the vortex finder length and the operating Reynolds number. In addition, the concentration field distribution of gas-liquid mixture within the proposed device is basically consistent with the gas volume fraction distribution given at present.

**Comment** *15. In the separation efficiency section, please explain first how to calculate gas separation efficiency using mathematical expression and liquid loss rate and then discuss the separation efficiency as a function of time and amplitude frequency, Hz.*

**Response to comment 15:** We thank the reviewer for the helpful comment. There are not enough variables to construct the function without changing the size structure and operating parameters of the hydrocyclone.
