# Supplementary material for: Study on flow field characteristics of gas-liquid hydrocyclone separation under vibration conditions
Source: PLoS One. 2024 Jul 12;19(7):e0307110. doi: 10.1371/journal.pone.0307110 (PMC11244842; doi:10.1371/journal.pone.0307110)
Supplement: Attachment — Submitted filename: Response to Reviewers-20240624.docx [file pone.0307110.s003.docx]

-------------------------------------------------------------------------------------------------------

**Response to the reviewer Comments: PONE-D-24-11282** **R1**

-------------------------------------------------------------------------------------------------------

Manuscript PONE-D-24-11282 R1

“Study on flow field characteristics of gas-liquid hydrocyclone separation under vibration conditions”

We would like to express our gratitude to the reviewers for their valuable feedback, which has contributed to further improving the quality of the paper. Below, reviewers’ comments are presented with *italics* to distinguish from our response. We also submit a Word copy of the revised manuscript in which important revisions are highlighted for easy identification.

This article must be strictly revised by considering the following comments.

**Comment** 1) *i.	Add a Table for model input parameters. Also mention convergence criteria.*

**Response to comment 1:** We thank the reviewer for the helpful comment. We have added the Table 3, which provides the model input parameters and convergence criteria mentioned in this article.

**Revision:** On Page 6, the last paragraph of section 2.4 "Boundary condition settings".

“

The input parameters and convergence criteria involved in the calculation of this article are shown in Table 3.

Table 3. Table for model input parameters and convergence criteria

| Type | Settings and valid values |
| --- | --- |
| **Fluid domain** | |
| Medium | water、oil、air |
| Density, kg/m^3^ | 998(water); 890(oil); 1.29(air) |
| Volume fraction | 0.65(water); 0.05(oil); 0.3(air) |
| **Structural domain** | |
| Medium | Structural Steel |
| Density, kg/m^3^ | 7850 |
| Elastic modulus, GPa | 210 |
| Poisson's ratio | 0.3 |
| **Operating parameter and convergence criteria** | |
| *n*, r*/*min | 100 |
| *T*, s | 0.59 |
| *f*, Hz | 1.7 |
| Excitation force *F*, N | 240 |
| Excitation displacement *X*, mm | *X*=240sin(10.6*time) |
| Time step, s | 0.005 |
| Residual convergence standard | 1×10^-6^ |

”

**Comment** 2) *ii.	Improve quality of Figures in particular the velocity vectors (Fig. 10) so that the flow pattern becomes clear.*

**Response to comment 2:** We thank the reviewer for the helpful comment. We have rechecked the relevant figures and replaced Fig. 10 with a higher quality one.

**Revision:** Fig. 10

“

Fig 10. Gas phase velocity vector diagram

”

**Comment** 3) *Further include a reference for the “PRESTO” method.*

**Response to comment 3:** We thank the reviewer for the helpful comment. We have added the.reference[26] for the “PRESTO” method, and updated the subsequent reference numbers.

**Revision:**

On Page 6, Paragraph 1 of the “2.4 boundary condition setting” section, Line 4, the revised text reads, “the pressure term[26],”.On Page 15, Paragraph 1 of the “References”, The newly added literature is “ Garrick D P, Rajagopalan R G. An Explicit Pressure-Based Algorithm for Incompressible Flows[C]. 22nd AIAA Computational Fluid Dynamics Conference. 2015: 3201.” The original reference numbers [26], [27], and [28] have been updated to [27], [28], and [29], respectively.
